# The efficacy of surgical decompression before 24 hours versus 24 to 72 hours in patients with spinal cord injury from T1 to L1 – with specific consideration on ethics: a randomized controlled trial

**DOI:** 10.1186/1745-6215-10-77

**Published:** 2009-08-24

**Authors:** Vafa Rahimi-Movaghar, Soheil Saadat, Alexander R Vaccaro, Seyed Mohammad Ghodsi, Mohammad Samadian, Arya Sheykhmozaffari, Seyed Mohammad Safdari, Bahram Keshmirian

**Affiliations:** 1 Research Centre for Neural Repair, Sina Trauma and Surgery Research Center, Tehran University Medical Sciences, Tehran, Iran; 2 Department of Epidemiology, Sina Trauma and Surgery Research Center, Tehran University Medical Sciences, Tehran, Iran; 3 Department of Orthopaedics and Neurosurgery, Thomas Jefferson University and the Rothman Institute, Philadelphia, PA, USA; 4 Department of Neurosurgery, Sina Trauma and Surgery Research Center, Tehran University Medical Sciences, Tehran, Iran; 5 Department of Neurosurgery, Shahid Beheshti University Medical Sciences, Tehran, Iran; 6 Department of Neurosurgery, Zahedan University Medical Sciences, Zahedan, Iran

## Abstract

**Background:**

There is no clear evidence that early decompression following spinal cord injury (SCI) improves neurologic outcome. Such information must be obtained from randomized controlled trials (RCTs). To date no large scale RCT has been performed evaluating the timing of surgical decompression in the setting of thoracolumbar spinal cord injury. A concern for many is the ethical dilemma that a delay in surgery may adversely effect neurologic recovery although this has never been conclusively proven. The purpose of this study is to compare the efficacy of early (before 24 hours) verse late (24–72 hours) surgical decompression in terms of neurological improvement in the setting of traumatic thoracolumbar spinal cord injury in a randomized format by independent, trained and blinded examiners.

**Methods:**

In this prospective, randomized clinical trial, 328 selected spinal cord injury patients with traumatic thoracolumbar spinal cord injury are to be randomly assigned to: 1) early surgery (before 24 hours); or 2) late surgery (24–72 hours). A rapid response team and set up is prepared to assist the early treatment for the early decompressive group. Supportive care, i.e. pressure support, immobilization, will be provided on admission to the late decompression group. Patients will be followed for at least 12 months posttrauma.

**Discussion:**

This study will hopefully assist in contributing to the question of the efficacy of the timing of surgery in traumatic thoracolumbar SCI.

**Trial Registration:**

RCT registration number: ISRCTN61263382

## Background

The role and timing of surgical decompression after an acute spinal cord injury (SCI) remains one of the most controversial topics pertaining to spinal surgery [1]. Despite an enormous amount of interest and research in SCI, the prognosis for neurologic recovery in patients with a severe SCI remains poor. Acute SCI remains an important cause of morbidity and mortality. Furthermore, the majority of patients with SCI are young, making the economic and societal impact immense [2].

Acute SCI involves both primary and secondary mechanisms of injury. The primary mechanism, usually caused by rapid spinal cord compression caused by bone displacement from a fracture-dislocation or burst fracture, is irreversible. It also initiates a cascade of secondary injury mechanisms, including ischemia, electrolyte derangements, and lipid peroxidation. Secondary injury is preventable and may be reversible [3].

The increased understanding of the pathophysiology of acute SCI has led to clinically relevant neuroprotective therapies to attenuate the effects of the secondary injury. The National Acute Spinal Cord Injury Studies (NASCIS II and NASCIS III) have shown a modest beneficial effect of high-dose methylprednisolone if given within 8 hours of injury in patients with SCI [4,5].

There is experimental evidence that persistent compression of the spinal cord is a potentially reversible form of secondary injury [6-10]. Dimar et al (1999) and Carlson et al (2003), Rahimi-Movaghar et al (2008) demonstrated in animal models that early decompression showed significantly better functional recovery and significantly smaller lesion volumes than delayed surgical intervention.

There are several prospective studies of surgical decompression in acute SCI [11-20]. In a non-randomized study, Papadopoulos et al (2002) evaluated 91 patients with acute cervical SCI to assess the feasibility and outcome of an immediate decompression treatment protocol. All patients, except 1, were admitted within 9 hours of their injury. The investigators reported that 39/66 patients in the protocol group had improvement following early surgery, including some presenting with a complete SCI, compared to 6/25 in the control group. They suggested that patients who had decompression with closed reduction alone (mean time to decompression 6.0 hours) had better neurologic outcomes than those requiring surgical decompression (mean time to decompression 12.6 hours) [13]. La Rosa et al (2004) performed a systematic review of all available studies published between 1966 and 2000. They concluded that early (<24 hours) surgical decompression in patients with incomplete injuries resulted in better neurologic outcomes than patients treated with either delayed decompression (>24 hours) or nonoperative treatment [21]. In contrast, several prospective studies have failed to document a beneficial effect of surgical decompression [11,12,19,20]. However, no study to date has truly examined in a systematic way a large cohort of randomized surgical patients who underwent decompression earlier than 24 hours. For example, although the study by Vaccaro et al (1997) was a prospective, randomized trial, 20 of the 62 patients were lost to follow-up, and "early" surgery was defined as being within 72 hours after SCI [12].

In contrast to the aforementioned studies of early decompression, Larson et al (1976) advocated operating a week or more after SCI to allow medical and neurologic stabilization of the injured patient [22]. This remains the practice in many institutions, particularly in light of early reports suggesting an increased rate of medical complications with early surgery (<5 days after SCI) [23]. Interestingly, a number of investigators have documented recovery of neurologic function after delayed decompression of the spinal cord (months to years) after the injury [24,25].

In the cases of incomplete thoracolumbar SCI, there are some criticisms for no surgery, because first, there is some evidence for effectiveness of decompression, second, instability could be harmful and increase neurologic deficit [26].

This RCT will try to evaluate the efficacy of surgical intervention in the setting of thoracolumbar spinal cord injury as has been done in previous retrospective studies [27,28]. Thus, we chose patients with acute spinal cord injury from T1 to L1. The clinical benefits of early or late surgery in improving neurologic recovery remains controversial because of the absence of well-designed and well-executed prospective, randomized controlled multi-centered trial.

In this study proposal, SCI patients with documented spinal cord compression will be assigned to either an early (<24 hrs) or later (24–72 hrs) decompression group (control group). The reason for choosing 24 hours as the cutoff for early decompression is that while NASCIS 2 & 3 trials [4,5] demonstrated benefit to patients treated within 8 hrs of their SCI, a multi-centered feasibility study conducted at SCI centers has demonstrated difficulty in instituting clinical treatment within an 8-hour time frame from the moment of injury [14]. This is a reflection of the time needed to transport a patient from the accident scene to a nearby hospital, then a Level I trauma center, and then to a MRI scanner to confirm the presence and location of spinal cord compression. Thus the 24-hour cutoff for early decompression was chosen as this represents an early decompression time frame that could realistically be applied to a clinical population of spinal cord injured patients.

In the last decade, many SCI clinical researchers have noted that RCTs are required in order to define the role of surgery in the management of acute SCI more properly [1,21,27,28], but withholding SCI patients who arrive early in the emergency room from early surgical decompression may not be acceptable by some surgeons.

The objectives of this study are to examine the benefits of neural decompression and its' relationship with neurologic improvement in regards to timing of intervention.

### Purposes

#### Primary purposes

To determine whether early or late surgical decompression is effective in neurological improvement in the setting of traumatic spinal cord injury from T1 to L1.

#### Secondary purposes

-To compare the neurological improvement in summed ASIA motor scores 12 months following a complete SCI in each of the early and late group of decompression

-To compare the neurological improvement in summed ASIA motor scores 12 months following an incomplete SCI in the early and late group following decompression

-To assess fusion success in the early and late group following decompression

-To assess sagittal alignment at the fracture level in the early and late group following decompression

-To compare the duration of acute hospitalization in the early and late group following decompression

-To compare the overall length of acute hospital stay in the early and late following decompression

-To compare the overall length of chronic hospital stay for rehabilitation [22] in each of the early and late decompression

-To assess the incidence of mortality in the early and late group following decompression

- To determine the frequency of complications in the early and late group following decompression

## Methods

### Design of Study

The study is intended to be a randomized clinical trial study.

### Sample size

The proportion of patients demonstrating at least a 5-point improvement in summed motor score in a previous study at our institution evaluating the timing of decompression in a complete SCI was 9% (*p*2) [28]. What we expect to be an intended (or at least acceptable) effect of the early decompression group is 33% of patients demonstrating at least a 5-point improvement in summed motor score (*p*1) [19]. To detect this difference with a sensitivity of 80% and an error probability of 5%, at least 149 patients per randomization group will be required using the following formula:



Considering only comparing the two groups of early and late decompression, our sample size should be at least 298 cases. To account for the possibility of 10% loss to follow-up, our estimated sample size is 328 cases.

### Participants

The diagnosis of spinal cord injury is based upon decreased superficial and deep sensation in the lower limbs, genitals and perinea area, decrease voluntary motor movement in lower limbs, and impaired urinary and anal sphincter control.

### Neurologic Evaluation

Motor and sensory examinations are performed at admission to the acute hospital, preoperatively, immediately after surgery, and 6 weeks, 6 months, 12 months, and at the most recent follow-up.

Outcome is measured by using the American Spinal Injury Association ASIA summed motor and sensory scores [29] and the NASCIS [4,5] system to score neurological status and motor and sensory function.

Patients are to be assigned an initial motor index score from 50 to 100 which includes manual muscle test scores of all key muscles, sensory examination of pin prick and touch, sacral and deep tendon reflexes, and muscle tone evaluation. Sensory level is recorded as the most caudal dermatomal level of bilateral intact sensation.

### Inclusion criteria

Participants are eligible if they are 18 years or older, have a spinal cord injury between T1 and L1 of traumatic etiology, are hemodynamically stable, have spinal cord compression on MRI and are between 0 hours and 24 hours post-injury.

### Exclusion criteria

Subjects with major and current psychiatric illness, who have significant traumatic brain injury associated with the spinal cord injury, major concurrent medical disease (including myocardial infarction within 3 months, uncompensated congestive heart failure, active systemic cancer, AIDS, diabetes mellitus), pre-injury major neurologic deficits or disease (e.g. stroke, Parkinson's disease, syringomyelia, Guilian-Barre), ankylosing spondylitis, penetrating injuries to the thoracolumbar, pregnant females, life-threatening injuries which prevent early decompression of the spinal cord, criminals, under indictment or incarceration, substance abuse, an ASIA Impairment Scale category of E, no cord compression on MRI, the presence of spinal shock, any cognitive deficit, unable to provide informed consent, and an injury which involves more than two adjacent vertebral levels. Selected participants are thoroughly informed of the trial and its attendant risks and asked to consent to be in one of two groups, those operated urgently in less than 24 hours from trauma or the late group who are operated and decompressed between 24 and 72 hours from trauma.

### Ethics

RCTs raise a number of ethical issues specifically related to the timing in the study design. If a patient has a potential chance of improvement by an early surgical decompression, it is ethically unacceptable to limit his/her access to this treatment. In both "early" and "late" decompression groups, everything including facilities and managements will be exactly the same except the time of decompression.

At first glance, the "difference" in chance of improvement between "early" and "late" groups seems unethical per se. During a group discussion we came to an agreement that what makes this RCT ethically unacceptable is not the above mentioned "difference" but withholding patients who could potentially benefit from early treatment. As the benefit of the timing of surgery is unclear and the timing of surgery in developed countries may be delayed as long as 4 or 5 days, delayed surgical intervention of greater that 24 hours is not an ethical concern. In developed countries, only 24% of SCI patients have access to a hospital setting that may provide early decompression surgery within the first 24 hours of injury [30]. This proportion is much less in developing countries because of lack of fundamental resources. [27]. We decided to set up a rapid response surgical team consisting of the same personnel providing regular care to SCI patients. When a SCI patient is referred to the hospital, he/she will randomly gets assigned into either the "early" or "late" treatment group. In the case of the "late" group, we will not interfere with the regular medical care process during the first 24 hours. If a patient comes to the hospital and he/she gets an "early" code, then the rapid response team will be summoned and the patient will be prepared for early surgery. We will not let the patient wait due to financial reasons, waiting lists, etc. All other interventions for rescue, resuscitation, hemodynamic stabilization, transport, imaging study, and surgical preparation will be provided in a standard way according to the guidelines of the hospital. Surgical, paramedical and nursing teams will be the same for both groups. Patients of the "early" group will benefit from the routine care provided for all other patients before and after surgery.

This design creates a "difference" in the potential chance of improvement among the two groups of patients by providing an "extra service" to one group rather than withholding the standard service from the other group. This solution may be a valid way of dealing with the ethical dilemmas associated with randomized controlled trials in SCI decompression.

All in all, to address the ethical problem in RCTs involving interference with the routine therapeutic process; it is suggested to provide extra services (in terms of speed or quality) to patients in clinical settings providing sub-optimal care.

The gap between medical care quality standards in developed and developing countries exits in many settings. This provides an opportunity for ethically acceptable RCTs in many health related fields which will result in a considerable addition to the medical science.

### Ethical approval

The trial approved by the ethics committees of the Sina Trauma and Surgery Research Center of Tehran University Medical Sciences and Shahid Beheshti University of Medical Sciences. The responsible ethics committees will monitor the trial.

### Informed consent

Written informed consent for this study will be obtained from the patient's authorized representative prior to the performance of any protocol-specific procedure. However, several of these assessments or tests may be performed as part of the patient's routine clinical evaluation (i.e. not specifically performed for this trial). The study will be conducted in accordance with the provisions of the Declaration of Helsinki, as amended in South Africa (1996).

### Allocation Concealment

A batch of sealed, opaque sequentially number envelopes will be provided to the supervising attending at each site. Whenever a patient is referred to a hospital, his/her name will be printed on the envelope and then the envelope will be opened. The patient will then receive the treatment identified within the envelope. The principal investigator will supervise commitment to the randomization process by reviewing the names printed on the opened envelopes and insuring that the patient receives the recommended treatment.

Unbiased examiners who are not involved in the management of the patients will perform the examinations. Thus, the design of the study is single-blinded.

### Randomization

Using blocked sample randomization generates the randomization list. The permuted block method of randomization for a block size of four was used. Complete and incomplete SCI patients each are allocated to the two groups, early (E) and late (L).

Randomization will be stratified by site. Separate blocked sequences will be allocated to each of 4 sites, therefore patients referred to a particular site will be randomized regardless of the treatment sequences of other sites.

### Control for Potential Confounding Factors

Prognostic baseline factors including age, sex, mechanism of injury, and associated injuries will be recorded at the time of enrollment in the study and will be controlled during the statistical analysis.

### Treatment

Emergency medical personnel will implement standard spinal immobilization and resuscitation techniques. All patients will receive intravenous methylprednisolone (30 mg/kg IV bolus over 15 minutes followed 45 minutes later by a 5.4 mg/kg/hr intravenous infusion over 23 hours if they arrive within the first 3 hours of injury and for 47 hours if they arrive between 3 and 8 hours post injury. [4] GI prophylaxis with antacids, H2 blockers or Proton Pump inhibitors will be prescribed.

### Early and late surgically decompressed group

The patients will be divided into two groups: Early (less than 24 hours from trauma) and late (between 24 and 72 hours following SCI). Because surgical technique varies according to the location of injury in the spine and the nature of the injury, surgeons are not restricted in their surgical technique. Radiographic parameters are evaluated on admission and post-operative images are done to assess the adequacy of decompression. Imaging studies are reported as soon as possible after the decompressive procedure but within a maximum of 7 days post treatment to assess the adequacy of the treatment.

Rehabilitation regimes start from the first day of trauma in all of the patients. Complications are identified and presented.

### Data Collection and Statistical analysis

Neurologic follow-up examinations are performed at routine in-hospital and outpatient follow-up visits by surgeons in each center. In all cases, the most recent follow-up data available are analyzed. In cases of delayed death, the last documented neurologic examination is used.

The following data is to be registered: age, sex, level of injury, the cause of trauma, time interval between trauma and admission, time interval between admission and operation, transition from the acute care hospital to rehabilitation, overall hospital (acute and rehabilitation) length of stay [23], complications such as pneumonia and atelectasis, superficial and deep sensory and motor index score and urinary and anal sphincter status on admission to the acute hospital; preoperative, postoperative and most recent motor scores at follow-up (at least 12 month follow-up).

### Descriptive and Explorative Statistics

Continuous variables including improvement of summed ASIA motor scores, summed ASIA sensory scores (light touch and pin prick) and the FIM scale will be managed with descriptive statistics (mean, SD, minimum, median, and maximum) and will be compared between treatment groups using the T-test.

Noncontinuous data including the occurrence and frequency of medical complications will be analyzed by the X^2 ^technique and organized in tables to include sample size, absolute and relative frequency.

The sample size was conducted on the percent of patients demonstrating an "improvement" in percent summed motor score. It is a dichotomous variable. This is our main method of analysis, but we also record ASIA motor score as a continuous variable to improve the power of our analysis.

In addition to univariable statistics, data will be analyzed by multivariable regression analysis to allow for statistical adjustment for important prognostic (confounding) factors.

Logistic regression is used to estimate the odds ratios and 95% confidence intervals. To perform data analysis, the SPSS-15 software applications are used. A P-value of less than 0.05 is considered statistically significant.

Factors suspected to be unbalanced between the groups would be accounted for by analysis of covariance.

### Clinical Significance

We will consider a result significant if there is a 5-point or greater improvement in the summed ASIA motor score beyond what would be expected analyzing recovery in the setting of spinal cord injury [31]. It means that anyone who demonstrates a 5 point improvement will be classified as "improved'. We intend to conduct logistic regression on those who improve using the ASIA scale.

Intention to treat analysis will be used.

### Risk/Benefit Discussion

Risks to patients who participate in either arm of the study are expected to be minimal as both the early and later treatment options are currently used in treating patients with thoracolumbar spinal cord injury. Although some authors [24,32] have warned against early surgery in patients with SCI due to an increased risk of medical complications or neurologic worsening, Wilberger [33] and Tator [20] have shown that patients undergoing early surgery with modern surgical techniques have similar or lower rates of complications compared to patients who have delayed surgery. The benefits to patients who participate in this study are unknown as it is not clear which of the groups may have a better neurologic or medical outcome. However, the benefit to society from the scientific answer to the question posed in this investigation is enormous.

#### Limitations

Four surgeons in four hospitals will participate in this study. This introduces the potential for bias in treatment selection. The neurological examination may be subject to inter and intra-observer variability as this is not performed by a single, independent observer. Interobserver and intraobserver reliability of the radiologic measurements will be assessed in our study [34].

## Conclusion

In conclusion, this study is aimed to study the effectiveness and indications of early and late surgical decompression in the setting of traumatic thoracolumbar SCI in a randomized format by independent, trained and blinded examiners [35]. This study can be a major step forward solving one of the controversial issues in the management of traumatic SCI.

This study provides an opportunity for ethically acceptable RCTs in many health related fields which will result in a considerable addition to the medical science.

## Competing interests

The authors declare that they have no competing interests.

## Authors' contributions

VRM conceived the trial design and was involved in subsequent adaptations and drafts of the manuscript. SMG, ARV, MS, SMS, BK and AS have contributed to adaptations from the original design. Soheil Saadat has contributed to statistical analysis and writing the draft for the ethical review board.

All authors read and approved the final manuscript.
